# Draft Genome Sequence of the Efficient Bioflocculant-Producing Bacterium *Paenibacillus* sp. Strain A9

**DOI:** 10.1128/genomeA.00131-13

**Published:** 2013-04-25

**Authors:** Bin-hui Jiang, Jin-liang Liu, Xiao-min Hu

**Affiliations:** College of Resource and Civil Engineering, Northeastern University, Shenyang, China

## Abstract

*Paenibacillus* sp. strain A9 is an important bioflocculant-producing bacterium, isolated from a soil sample, and is pale pink-pigmented, aerobic, and Gram-positive. Here, we report the draft genome sequence and the initial findings from a preliminary analysis of strain A9, which is a novel species of *Paenibacillus*.

## GENOME ANNOUNCEMENT

*Paenibacillus* sp. strain A9, which is designated *Paenibacillus* based on its 16S rRNA gene sequence and can produce bioflocculant with high flocculating activity, was screened from soil near a peach tree in Liaoning province, China. It is a pale pink-pigmented, aerobic, Gram-positive, bioflocculant-producing bacterium that represents a species in the family *Paenibacillaceae*. A bioflocculant, MBFA9, was produced from strain A9 and had a good flocculating capability; it could achieve a flocculating rate of 99.6% for kaolin suspension at a dosage of only 0.1 ml/liter (1). Over the past couple of years, the genome sequences of *Paenibacillus* genus members *Paenibacillus alvei* DSM 29 (2), *Paenibacillus polymyxa* Sc2 (3), *Paenibacillus mucilaginosus* 3016 (4), *Paenibacillus vortex* V453 (5), and *Paenibacillus terrae* HPL-003 (6) have been published.

The genome sequence of A9 was determined by Shanghai Majorbio Bio-pharm Technology Co., Ltd. (Shanghai, China) using Solexa paired-end sequencing technology (7). A total of 4,387,020 paired-end reads (300-bp library) were generated to reach a 161.4-fold depth of coverage with Illumina/Solexa Genome Analyzer IIx (Illumina, San Diego, CA). Open reading frames (ORFs) were identified by using Glimmer. All predicted ORFs were then annotated by BLAST and KEGG.

There were a total of 4,284 putative open reading frames (with an average size of 1,113 bp) according to Glimmer. The obtained genome sequence of A9 consists of 92 contigs of 5,489,018 bp and has an average G+C content of 46.55%. The draft genome (about 4 Mbp) contains 92 contigs, which can be assembled into 47 scaffolds. The scaffold N_50_ is 203,315 bp. Automatic gene annotation was carried out by the NCBI Prokaryotic Genomes Automatic Annotation Pipeline (PGAAP) (http://www.ncbi.nlm.nih.gov/genomes/static/Pipeline.html), which was followed by manual editing. A total of 1,689 proteins were assigned to Clusters of Orthologous Groups (COG) families. Fifty-seven tRNA genes for 19 amino acids (lacking Asp) and one 16S-23S-5S rRNA operon were identified. The proteins associated with carbohydrate transport and metabolism (COG initial, G) were the most abundant group of COG (209 ORFs, 12.4%), followed by those associated with amino acid transport and metabolism (E, 168 ORFs, 10.0%) and transcription (K, 145 ORFs, 8.6%). The A9 genome sequence and its curated annotation are important assets for better understanding the physiology and metabolic potential of *Paenibacillus* sp. and will open up new opportunities in the functional genomics of this species.

A more specific analysis of strain A9 will be reported in a future publication.

### Nucleotide sequence accession number. 

The draft genome sequence of *Paenibacillus* sp. A9 is available in GenBank under the accession no. AOIG00000000.
